# Gene Expression Networks Across Multiple Tissues Are Associated with Rates of Molecular Evolution in Wild House Mice

**DOI:** 10.3390/genes10030225

**Published:** 2019-03-18

**Authors:** Katya L. Mack, Megan Phifer-Rixey, Bettina Harr, Michael W. Nachman

**Affiliations:** 1 Department of Integrative Biology and Museum of Vertebrate Zoology, University of California, Berkeley, CA 94720, USA; 2 Department of Biology, Monmouth University, West Long Branch, NJ 07764, USA; mphiferr@monmouth.edu; 3 Max-Planck-Institute for Evolutionary Biology, Plön 24306, Germany; bettina.harr@mac.com

**Keywords:** co-expression, gene regulation, house mice

## Abstract

Interactions between genes can influence how selection acts on sequence variation. In gene regulatory networks, genes that affect the expression of many other genes may be under stronger evolutionary constraint than genes whose expression affects fewer partners. While this has been studied for individual tissue types, we know less about the effects of regulatory networks on gene evolution across different tissue types. We use RNA-sequencing and genomic data collected from *Mus musculus domesticus* to construct and compare gene co-expression networks for 10 tissue types. We identify tissue-specific expression and local regulatory variation, and we associate these components of gene expression variation with sequence polymorphism and divergence. We found that genes with higher connectivity across tissues and genes associated with a greater number of cross-tissue modules showed significantly lower genetic diversity and lower rates of protein evolution. Consistent with this pattern, “hub” genes across multiple tissues also showed evidence of greater evolutionary constraint. Using allele-specific expression, we found that genes with cis-regulatory variation had lower average connectivity and higher levels of tissue specificity. Taken together, these results are consistent with strong purifying selection acting on genes with high connectivity within and across tissues.

## 1. Introduction

Understanding the forces that govern genetic and phenotypic variation within and between species is an enduring problem in evolutionary biology. The number of interactions between genes and the phenotypic consequences of these interactions may be important determinants of evolutionary constraint [1,2]. For example, a gene with many interactions in a gene regulatory network common across cells may be more pleiotropic than genes in the periphery of that network, or genes with tissue-specific expression [3,4]. Such highly connected genes are expected to be under strong negative selection, as any change to these genes could affect their downstream partners [3]. One approach to studying relationships between genes across the genome is to create gene co-expression networks, summarizing relationships between genes based on their coordinated expression across samples. The relationship between one gene and all other genes can be assessed based on the strength of connection between that gene and others in a network. Genes whose expression is more highly correlated with other genes in the network are thus more “connected” within a co-expression network. Gene co-expression is of biological interest as co-expressed genes are expected to be controlled by the same transcriptional regulatory program or otherwise be functionally related. Gene co-expression network analysis has been used to study co-expressed gene sets, compare patterns across tissues [5], between species [6,7,8], and to identify sets of functionally related genes associated with quantitative or disease phenotypes [9,10,11,12].

A general feature of co-expression networks is that there are a small number of highly connected genes and many genes with very few connections [13]. The few highly connected genes are expected to show higher levels of pleiotropy compared to genes with fewer connections, and consequently are predicted to be more constrained both in terms of changes in gene expression and in protein sequence. Consistent with this, a number of studies have found that more connected genes exhibit lower genetic diversity and lower rates of molecular evolution [14,15,16]. These findings parallel what has been seen in protein–protein interaction networks, where genes encoding proteins with more protein–protein interactions have been shown to evolve more slowly than genes with fewer interactions (e.g., [1,2]).

The interplay of co-expression network topology and gene expression across tissues has received less attention. However, differences in co-expression networks between tissues may affect sequence evolution. All cells carry out a combination of common and tissue-specific processes associated with their unique phenotypes, and tissues contain a collection of different cell types. Consequently, genes that are highly connected in one tissue type may be more peripheral in others. A simple prediction that follows from this is that genes that are highly connected in more tissues should, on average, be less tolerant of sequence variation and evolution, as these genes may be more pleiotropic. Comparisons of co-expression networks across tissues have been used to characterize network topology across different tissues [5,17], but how the properties of these networks affect sequence evolution remains unexplored.

House mice are a biomedical model system and have extensive genomic resources (*Mus musulus domesticus*) [18,19], making them a powerful system for studying co-expression networks. To investigate the relationship between cross-tissue co-expression networks and molecular evolution, we constructed co-expression networks for 10 tissue types in 21 progeny of mice collected from natural populations. We used these data to compare co-expression network topology between tissues, identify tissue specific expression and local regulatory variation, and associate these components of gene expression variation with sequence variation and evolution.

## 2. Materials and Methods

### 2.1. Expression Data

RNA-sequencing (RNA-seq) data were downloaded from Harr et al. [20]. These samples correspond to lab-born progeny of *M. m. domesticus* collected from Germany, Iran, and France, with up to 10 tissue types per individual (muscle, thyroid, brain, testis, spleen, liver, gut, heart, lung, kidney) (File S1). Detailed descriptions of sample locations and breeding design can be found in Harr et al. [20] (http://wwwuser.gwdg.de/~evolbio/evolgen/wildmouse/). To avoid sampling relatives, individual mice were collected between 500 m–1 km apart, covering an area of no more than 50 km radius for each of the three populations. Samples for DNA and RNA-sequencing were obtained from the first or second generation of out-breeding in an animal facility and are expected to represent full wild-type variation. Individuals used for RNA-seq were age-matched males (10–12 weeks of age). We downloaded RNA-seq reads mapped with Tophat2 [21] to the mm10 reference genome [20]. We then counted reads that mapped to exonic regions using HTSeq-count [22] based on the Ensembl GRCm38 annotation.

### 2.2. Co-Expression Analysis

Three individuals were removed because of relatedness (first- or second-degree relatives), leaving 21 individuals for co-expression analyses. Samples that were tissue-specific outliers (3) were identified through a principal component analysis and removed from subsequent analyses of that tissue type (see File S1 for a list of samples included in this analysis for each tissue type). This resulted in 188 samples for downstream analysis. For individual co-expression analyses, genes with fewer than 20 reads on average per tissue were removed. Gene expression was then quantile-normalized and then concurrently corrected for known and unknown covariates (first 5 principal components of genotype data to account for population structure and 10 hidden confounders), which are known to explain variation in gene expression, using a Bayesian approach implemented in the program PEER [23,24]. Accounting for hidden factors and other confounders reduces the influence of this variation on downstream analyses, variation that can obscure true signals or generate false signals due to covariance [25]. Principle components are frequently used to correct for population structure in gene expression data (e.g., [26,27]). The R program SNPrelate was used to perform principal component analyses on genotype data [28]. The program Weighted Gene Co-expression Network Analysis (WGCNA) was then used to construct co-expression networks for all tissue types for all individuals, following WGCNA protocols [29]. In short, we first constructed a gene co-expression network, represented by an adjacency matrix, which denotes co-expression similarity between pairs of genes among different individuals, for each tissue. Then, modules were identified using unsupervised clustering. Dissimilarly between clusters is measured based on topological overlap and defined by cutting branches off the dendrogram [29,30]. Modules are then arbitrarily assigned colors for identification. Each module is summarized by a representative eigengene, or the first principal component of the module. Each gene’s total connectivity within a tissue was then retrieved using the command *intramodularConnectivity*.

**Figure 1:**
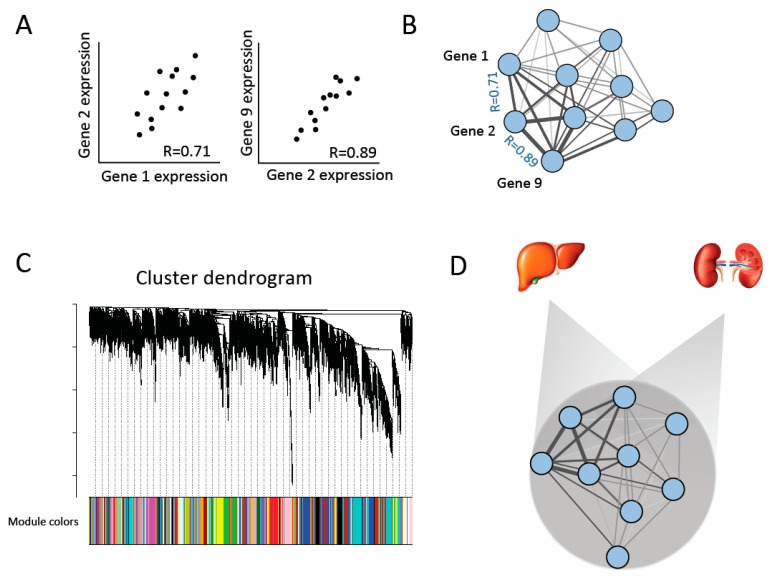
Constructing gene co-expression networks [29]. (**A**) Co-expression similarity is compared between pairs of genes among individuals in order to build (**B**) a co-expression network of all genes. (**C**) Co-expression modules are identified and defined by hierarchical clustering and cutting branches off the dendrogram. Modules are then assigned colors for identification. (**D**) Consensus networks across each pair of tissues are created to identify co-expression modules that are conserved across tissues (consensus modules) [31].

To identify co-expression modules that were conserved across tissues, we also restricted our analysis to genes that were expressed in every tissue (with a minimum average read depth of 50 reads/sample; 10,780 genes) and created consensus networks between each possible pair of tissues (45 comparisons) following WGCNA protocols [29,31]. The consensus network is a single network arising from two tissues (Figure 1D). In this way, a consensus network analysis differs from analyses where modules are identified in one “reference” network and preservation of these modules is studied in other networks (e.g., [32]). Instead, consensus modules are expected to represent co-expression patterns conserved across tissues [29,31].

As in a single network analysis (see above), consensus modules are defined based on unsupervised clustering and arbitrarily assigned colors for identification.

### 2.3. Tissue Specificity

To compare gene expression across tissue types and identify genes with tissue specific expression, mapped reads were downsampled across samples/tissue types to account for differences in average library size between individual samples. For our analysis of tissue specificity, genes with fewer than an average of 50 reads across all samples were discarded. Tissue specificity was subsequently defined as in Sonawane et al. [17]:

$$S_{j}^{\left(t\right)}=med\left(e_{j}^{\left(t\right)}\right)-med\left(e_{j}^{\left(all\right)}\right)-IQR\left(e_{j}^{\left(all\right)}\right)$$

where the specificity (*S*) of gene *j* in tissue *t* corresponds to (the median (*med*) expression (*e*) of the gene in that tissue (*t*)—the median expression of the gene in all tissues (*all*))—interquartile range (*IQR*) of expression of that gene across all tissues. A gene’s highest *S* value across all 10 tissues was designated *S*_max._ Genes in a tissue for which *S* > 2 were considered tissue-specific. Under this definition, genes can be tissue specific in more than one tissue. The number of tissues for which a gene was considered tissue-specific is the gene’s multiplicity value. For example, a gene with *S* > 2 in three tissues has a multiplicity of three. A total of 4902 genes were found to be tissue specific in just one tissue type, meaning these genes have a multiplicity of one.

### 2.4. Allele-Specific Expression

To identify allele-specific expression, we downloaded genome-wide single nucleotide polymorphism (SNP) calls from Harr et al. [20] for these individuals, filtering variants based on the “PASS” flag. Two individuals (132 and IR122) did not have corresponding genomic data and were not included in this analysis. To test for allele-specific expression in each tissue, RNA-seq reads mapped to the reference and alternative allele for heterozygous sites were counted using GATK ASEReadCounter [33]. Heterozygous sites with fewer than 20 mapped RNA-seq reads supporting the reference and the alternative allele were discarded. Allele-specific expression was then called as described in [34]. The number of single-nucleotide polymorphism (SNPs) that could be tested in each tissue is listed in Table S1, corresponding to a total of 15,390 genes across all tissue types. We retained the variants with the lowest *p*-values per gene and then performed a false-discovery rate correction using R’s *p.adjust* (Table S2).

### 2.5. Measures of Sequence Evolution

Estimates of dN (nonsynonymous substitutions per nonsynonymous site) and dS (synonymous substitutions per synonymous site) between mouse and rat were downloaded from Ensembl (GRCm38) [35]. We also calculated dN and dS between *M. m. domesticus* and *Mus caroli.* Expression level, expression variation, and connectivity were compared to dN/dS for individual genes to quantify the effects of gene expression on rates of protein evolution. Similar results were obtained in comparisons between mouse and rat as in comparisons between *M. m. domesticus* and *Mus caroli.* (Table S3). Given the similarity of the results, only dN/dS comparisons between mouse and rat are reported in the text. SNP density was estimated based on genome-wide SNP calls from Harr et al. [20], counting SNPs that fell within the boundaries of each gene and correcting for the length of a gene using gene start and stop annotations downloaded from Ensembl (GRCm38). Gene start and end coordinates correspond to the outermost transcript start and end coordinates, meaning these regions contain both exonic and intronic sites.

### 2.6. Enrichment Analyses

Tests for enrichments of mutant phenotypes were done using modPhEA [36]. All Gene Ontology (GO) category enrichment analyses were performed with PANTHER [37].

### 2.7. Protein Interaction Networks

Predicted protein networks were downloaded from STRING (v10) [38] for *M. m. domesticus*. STRING predicts interactions based on various sources of data, including experimental, text-mining, and databases [39]. Interactions were filtered for “high confidence” interactions (>0.7; [38]).

### 2.8. Variant Annotations

Variants were annotated (e.g., synonymous, nonsynonymous) using the Ensembl Variant Effect Predictor [40].

We analyzed genome-wide expression data generated by Harr et al. [20] for 188 tissue samples from 21 male *M. m. domesticus*. These samples correspond to 10 different tissue types (muscle, thyroid, brain, testis, spleen, liver, gut, heart, lung, kidney) collected from lab-born progeny of wild house mice of diverse genotypes captured in Iran (*n* = 6), France (*n* = 7), and Germany (*n* = 8), and raised in a common environment (see File S1).

## 3. Results

### 3.1. Properties of Gene Connectivity within and Across Tissues

To characterize properties of gene connectivity within and across tissue, we used WGCNA [29] to construct co-expression networks, identify co-expression modules, and estimate gene connectivity across all individuals. Gene expression was quantile-normalized for each tissue type and then corrected for hidden cofounders and population structure (see methods). Individuals from all three populations were then used to construct a gene co-expression network for each tissue type.

In a gene co-expression network analysis, the expression of each pair of genes is compared across samples to create a co-expression network. A gene’s connectivity is defined as the sum of connection strengths between a focal gene and all other genes in a network. Genes with similar expression patterns can then be grouped into co-expression modules (see methods) [29] (Figure 1).

First, we investigated general properties of co-expression network topology within and across tissue types. Consistent with previous studies with single tissues [15], we found a significant positive correlation between connectivity and gene expression level for each tissue type (Spearman’s rank correlation, Table S4). Gene connectivity was also correlated between different tissue types (Spearman’s rank correlation, Table S5), with correlation coefficients ranging between 0.06–0.35 in pairwise comparisons between tissues. Testis, brain, and spleen showed the lowest average correlation coefficients in pairwise comparisons between these and other tissues.

To investigate co-expression relationships across tissues, we also used WGCNA to identify modules that are shared across two tissue types, known as consensus modules (Figure 1) [29,31]. To identify consensus modules, we restricted our analysis to genes expressed across all tissue types (10,780 genes) and built co-expression networks for each pair of tissues (45 comparisons total) (see methods). We then counted the number of consensus modules with which each gene was significantly associated. For example, a gene that is significantly associated with a co-expression module between every pair of tissues would be found in 45 consensus modules, whereas a gene that is only found in a consensus module between the liver and spleen would be found in one consensus module. Average expression was significantly positively associated with the number of consensus modules in which a gene was found (Spearman’s rank correlation, *rho* = 0.88, *p* < 2.2 × 10^−16^), as was average gene connectivity across tissues (Spearman’s rank correlation, *rho* = 0.79, *p* < 2.2 × 10^−16^). We also observed a significant, but weaker, negative association with tissue-specificity (see below) (Spearman’s rank correlation, *rho* = −0.088, *p* = 1.17 × 10^−15^), where genes that had higher tissue-specificity values were found in fewer consensus modules.

### 3.2. Tissue Specific Expression and Connectivity

Differences in connectivity between tissues are expected to be a consequence of tissue-specific expression patterns. To identify genes with tissue-specific expression, we compared the expression of each gene across the 10 tissue types included in this study and estimated each gene’s specificity (*S*) for a tissue, based on the gene’s expression level and variance across all tissues (see methods) (Figure S1) [17]. Consistent with other studies on tissue-specificity [41,42], the testis had the greatest number of genes with tissue-specific expression, followed by the brain.

Tissue-specific genes were found to have higher connectivity on average within their tissue when compared to non-tissue specific genes (permutation tests, all comparisons *p* < 0.0001), but we also observed a weak negative correlation between a gene’s level of tissue specificity (*S*_max_) and connectivity across all tissues (Spearman’s rank correlation, *rho* = −0.035, *p* = 0.0014) (Figure S2). Using annotations from the DBD transcription factor prediction database [43], we found that tissue-specific genes were significantly less likely to encode transcription factors (χ^2^ test, *p* = 0.0069, Odds ratio = 0.80). While there are very few transcription factors that were tissue-specific, the tissue-specific transcription factors were enriched for tissue-specific mutant phenotypes in some tissues (Table S6). For example, tissue-specific brain transcription factors were enriched for mutant phenotypes related to abnormal brain size (*q* = 1.725 × 10^−4^), abnormal cerebellar cortex morphology (*q* = 1 × 10^−3^), and abnormal brain weight (*q* = 1.725 × 10^−4^), compared to the background set of genes expressed across tissues.

### 3.3. Relationship between Regulatory Variation and Connectivity

Previous studies have found that genes with local (*cis*-) regulatory variation also show lower average connectivity in gene expression networks [15,16]. One interpretation of this observation is that genes in the periphery of a network are more tolerant of local regulatory variation. To investigate the relationship between connectivity and regulatory variation in house mice, we identified genes with allele-specific expression in each tissue type. Allele-specific expression, the difference in expression between parental alleles, can be used to identify *cis* acting epigenetic or genetic variation in heterozygous individuals [44]. In each tissue, we tested exonic heterozygous sites for differences in expression between parental alleles (see Methods) (Table S1). We identified 4146 genes with allele-specific expression across all 10 tissue types (False-discovery rate < 0.1; Table S2), many of which (28.48%) showed allele-specific expression in more than one tissue-type.

We then tested whether genes with allele-specific expression showed lower average connectivity within a tissue. As the power to detect allele-specific expression increases with expression level [45] (Figure S3), connectivity scores were adjusted for average expression level within each tissue (see methods). We found that in all tissues, genes with *cis*-regulatory variation had lower average connectivity than genes without *cis*-regulatory variation (permutation tests, all comparisons *p* < 0.0001). We also found that genes with allele-specific expression had higher levels of tissue-specificity on average (permutation test, *p* < 0.0001; *S*_max_ adjusted for average expression level across tissues).

Genes with *cis*-regulatory variation may have lower average connectivity if genes with higher connectivity are under stronger purifying selection and thus less tolerant of *cis*-regulatory variation. Consistent with this, we find that genes with allele-specific expression in any tissue have higher dN/dS values (Mann–Whitney U, *p* = 0.03). We also downloaded predicted protein interaction data from STRING [39] and found that genes with allele-specific expression encoded proteins that have fewer interacting partners on average (Mann–Whitney U, *p* < 2.2 × 10^−16^). Finally, we found that genes with allele-specific expression were less likely to encode transcription factors (χ^2^ test, *p* < 0.0001). This was also observed for transcription factors that were considered tissue-specific (χ^2^ test, *p* < 0.0001).

### 3.4. Relationship between Connectivity and Sequence Evolution

**Figure 2:**
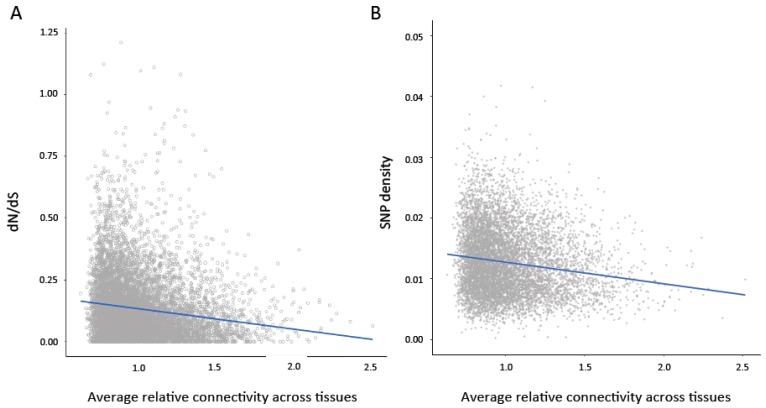
(**A**) Average connectivity across tissues is significantly negatively correlated with dN/dS ratio (Pairwise Spearman’s rank correlation *rho* = −0.18, *p* < 0.0001; Partial Spearman *rho* = −0.045, *p* < 0.0001). (**B**) Average connectivity across tissues is significantly negatively correlated with single nucleotide polymorphism (SNP) density (Pairwise Spearman’s rank correlation *rho* = −0.16; *p* < 0.0001, Partial Spearman *rho* = −0.09, *p* < 0.0001).

To examine the relationship between evolutionary constraint and characteristics of gene expression, we performed pairwise tests between aspects of gene expression across tissues (average connectivity, average expression level, and variance in expression and connectivity across tissues) and measures of sequence variation (SNP density) and protein evolution (dN/dS ratio) (Table 1). To control for the relationship between these measures and different aspects of gene expression, we then performed partial Spearman correlations between properties of gene expression (listed in Table 1) and sequence evolution. In contrast to pairwise tests, partial correlations measure the degree of association between two variables when other variables are removed to control for the confounding relationship between multiple variables. We found that average connectivity and average expression level across tissues showed highly significant negative associations with dN/dS ratio (Figure 2A) and SNP density (Figure 2B) (Table 1). We also performed 1000 permutations in which the relationship between the predictors and dN/dS ratio and SNP density was randomized. None of the correlations in the permutated datasets were more extreme than the observed partial correlations. Despite the broad patterns shown in Figure 2, we note that there are some genes with high levels of constraint that show low connectivity (bottom left corner of Figure 2A,B). In contrast, there were no genes with very low levels of constraint and very high connectivity.

**Figure 3:**
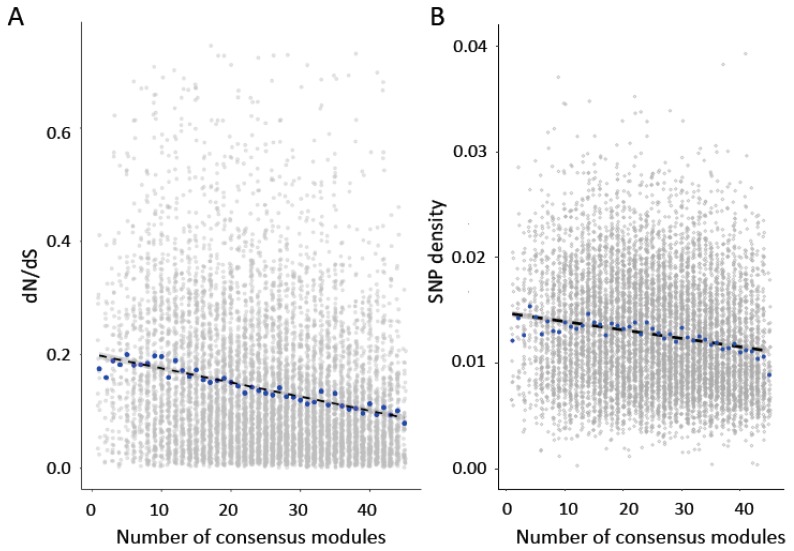
(**A**) Genes in more consensus modules show significantly lower dN/dS values (Pairwise Spearman’s rank correlation *rho* = −0.22, *p* < 2.2 × 10^−16^; Partial Spearman *rho* = −0.11, *p* < 2.2 × 10^−16^). (**B**) Genes in more consensus modules also show significantly lower SNP density (Pairwise Spearman’s rank correlation *rho* = −0.16, *p* < 2.2 × 10^−16^; Partial Spearman *rho* = −0.039, *p* = 0.00036). Blue points indicate median values.

Modules that are preserved across tissues are expected to have functions that are common across tissues [5]. To assess whether the preservation of module relationships across tissues was also associated with rates of sequence evolution, we asked whether genes found in a greater number of consensus modules between pairs of tissue types showed greater sequence conservation. We predicted that genes that were found in more modules across tissues would show greater sequence constraint, as these genes may also show higher levels of pleiotropy. Consistent with prediction, we found that dN/dS (Figure 3A; Spearman’s rank correlation, *rho* = −0.22, *p* < 2.2 × 10^−16^) and SNP density (Figure 3B; Spearman’s rank correlation, *rho* = −0.16, *p* < 2.2 × 10^−16^) were significantly negatively correlated with the number of consensus modules in which a gene was found. As in the previous analysis, we also performed a partial Spearman correlation to account for average expression level, expression variance, gene connectivity, and variance in connectivity across tissues. We found that the association between dN/dS ratio (Partial Spearman correlation, *rho* = −0.11, *p* < 2.2 × 10^−16^) and SNP density (Partial Spearman correlation, *rho* = −0.039, *p* = 0.00036) were still significant when accounting for these variables. In 1000 permutations in which the relationship between pair number and dN/dS ratio or SNP density was randomized, no correlation was more extreme than that observed for the dN/dS ratio and only one permutation was more extreme than that observed for SNP density.

To better understand the observed associations between SNP density and these measures, we filtered our SNP set to only include coding variants. We found that both the number of nonsynonymous and the number of synonymous polymorphisms within genes were negatively correlated with a gene’s average connectivity (nonsynonymous: Spearman’s *rho* = −0.078, *p* = 5.96 × 10^−12^; synonymous: Spearman’s *rho* = −0.072, *p* = 1.49 × 10^−6^) as well as with the number of consensus modules a gene was associated with (nonsynonymous: Spearman’s *rho* = −0.091, *p* < 2.2 × 10^−16^; synonymous: Spearman’s *rho* = −0.07, *p* = −1.83 × 10^−10^). These patterns may be a consequence of either direct selection against deleterious variants and/or background selection against linked deleterious variants. While most synonymous mutations are expected to be neutral, there are some examples of deleterious synonymous mutations that affect protein folding [46].

### 3.5. Constraint on Cross-Tissue Hub Genes

Co-expression analyses have been widely applied to identify “hub” genes, or genes whose expression is highly correlated with their expression module. Hub gene analysis has also become a popular method for identifying genes whose expression is related to variation in quantitative traits [9] or disease phenotypes (e.g., [10,11,12]). As hub genes represent the genes most highly associated with their module’s expression, we expected genes that were annotated as hubs in more than one tissue to be involved in upstream processes common across cells and therefore show greater sequence constraint.

Using the individual co-expression networks we created for each tissue, we first identified hub genes by estimating each gene’s module membership. Each gene’s module membership was estimated based on the correlation between that gene’s expression and the expression of the module eigengene [29]. Genes where module membership was greater than 0.8 were considered “hub genes” for subsequent analyses, a cut-off selected because of its usage in previous studies (e.g., [11]). Consistent with what has been seen in human populations [17], we found that genes that encode transcription factors were more likely to be hub genes (χ^2^ test, *p* = 0.0002, Odds ratio = 1.25).

We then compared hub genes across tissues. We found that a large proportion of the hub genes we identified in our analysis are unique to one tissue type (61%), and only 9.2% of these genes were annotated as hubs in 3 or more tissues (Figure S1). Consistent with the idea that cross-tissue hub genes represent genes with essential biological functions, we also found that genes that were identified in hubs in 3 or more tissues were highly enriched for mutant phenotypes related to mortality/aging (*q* = 2.93 × 10^−12^; including significant enrichment of the mortality/aging subcategories abnormal survival, preweaning lethality, prenatal lethality, and embryonic lethality), abnormal cell physiology (*q* = 5.66 × 10^−5^), and abnormal homeostasis (*q* = 1.98 × 10^−4^). These genes were also enriched for several GO terms, including positive regulation of biological process (*q* = 1.03 × 10^−26^) and regulation of cellular processes (*q* = 3.01 × 10^−22^). Genes annotated as hubs in just two tissues were also significantly enriched for mutant phenotypes related to mortality/aging, but this enrichment was less significant (*q* = 0.01).

**Figure 4:**
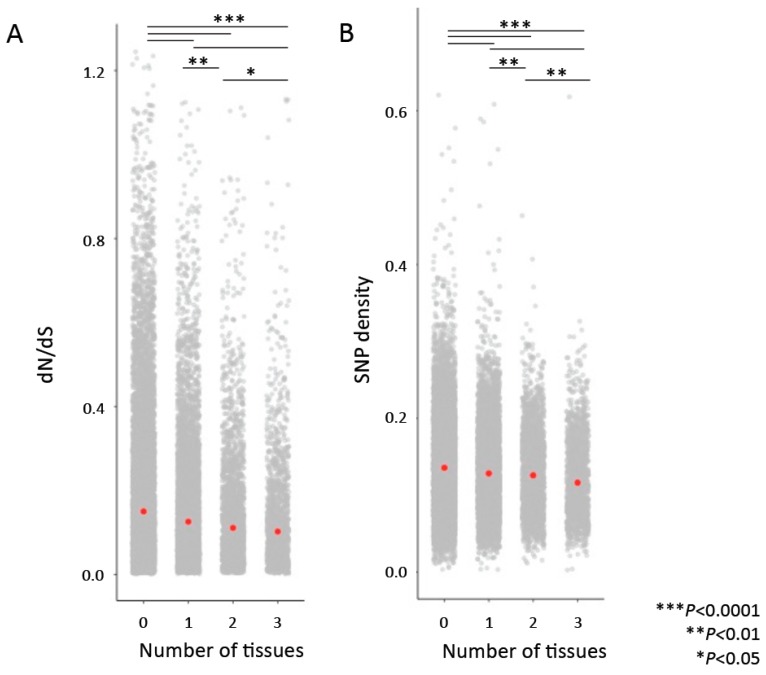
Genes that are “hubs” in more tissues are associated with lower dN/dS values (**A**) and lower SNP density (**B**). Comparisons were performed with permutation tests. Red points indicate median values.

Parallel to the previous analyses, where we asked whether more highly connected genes or genes found in a greater number of cross-tissue modules were more constrained, we also asked whether genes annotated as hubs in more tissue types were under greater evolutionary constraint by comparing the dN/dS ratios and SNP densities for genes identified as hubs in no tissues, one tissue (*n* = 6632), two tissues (*n* = 2532), and three or more tissues (*n* = 1001). We found that genes identified as hubs in more tissues showed lower average dN/dS and SNP density (Figure 4).

## 4. Discussion

Here, we have used natural populations of house mice to characterize co-expression networks for 10 tissue types and associate components of gene expression variation with sequence variation and evolution. Genes with higher connectivity across tissues showed significantly lower genetic diversity and lower rates of protein evolution. We also found that genes in more consensus modules across tissues show significantly lower genetic diversity and lower rates of protein evolution. The association between these measures and reduced genetic diversity may be a consequence of selection against deleterious variants and/or purifying selection acting on deleterious mutations at linked sites (background selection) [47]. Genes that were hubs across more tissues likewise showed evidence of evolutionary constraints and were significantly enriched for mutant phenotypes related to mortality and aging. Finally, we found that genes with allele-specific expression had lower connectivity on average, higher dN/dS values, and fewer connections in protein–protein interaction networks. Altogether, this suggests that genes with allele-specific expression may be less constrained. In this regard, we speculate that regulatory variation at peripheral genes may provide variation that can act as a substrate for adaptive evolution. Altogether, our results suggest that gene connectivity is an important determinant of evolutionary constraint.

## Figures and Tables

**Table 1:** Spearman’s rank correlation coefficient between gene expression-related measures and sequence evolution.

	dN/dS		SNP Density	
Variable	Pairwise ^1^	Partial ^2^	Pairwise	Partial
Average expression level across tissues	−0.26 ***	−0.15 ***	−0.15 ***	−0.14 ***
Expression IQR across tissues	−0.22 ***	0.042 **	−0.05 ***	0.17 ***
Average connectivity across tissues	−0.18 ***	−0.045 ***	−0.16 ***	−0.09 ***
Connectivity IQR across tissues	−0.12 ***	0.04 **	−0.11 ***	−0.04 ***

^1^ Pairwise correlations measure the correlation between the variable and dN/dS or SNP density. ^2^ Partial correlations measure the association between the variable and dN/dS or SNP density when other variables are accounted for. Interquantile range (IQR), where IQR = Quantile 3 − Quantile 1. *** *p* < 0.0001; ** *p* < 0.001. SNP: Single-nucleotide polymorphism. dN/dS: Nonsynonymous to synonymous substitutions rate ratio.

## Supplementary Materials

The following are available online at https://www.mdpi.com/2073-4425/10/3/225/s1, Figure S1: (A) The number of genes in each tissue that were classified as tissue-specific. In orange are genes that encode transcription factors. (B) The number of hub genes that are found across different numbers of tissues, Figure S2: (A) Average expression across tissues and S_max_ are negatively correlated. (B) Connectivity across tissues is negatively correlated with S_max_, Figure S3: Genes for which we could detect allele-specific expression have higher expression on average (permutation test, *p* < 0.0001), Table S1: Number of genes with a SNP that could be tested for allele-specific expression (ASE), Table S2: Number of genes with allele-specific expression, Table S3: Spearman’s rank correlation coefficient between gene expression-related measures and dN/dS between *M. m. domesticus* and *M. caroli*, Table S4: The relationship between gene expression and connectivity within tissues, Table S5: Pairwise comparisons of gene connectivity between tissues (Spearman’s rank correlation, rho), Table S6: Tissue-specific transcription factors are enriched for tissue-specific mutant phenotypes, File S1: Samples included in co-expression analysis after filtering.
