# Supplementary material for: Gene Expression Networks Across Multiple Tissues Are Associated with Rates of Molecular Evolution in Wild House Mice
Source: Genes (Basel). 2019 Mar 18;10(3):225. doi: 10.3390/genes10030225 (PMC6470930; doi:10.3390/genes10030225)
Supplement: Supplementary file 1 [file genes-10-00225-s001.zip › supplementary material.docx]

**Supplemental material**

**Table S1.** Number of genes with a SNP that could be tested for allele-specific expression (ASE).

| Tissue | Number of samples | Number of genes that could be tested for ASE |
| --- | --- | --- |
| Spleen | 18 | 8,389 |
| Brain | 20 | 7,973 |
| Heart | 20 | 6,351 |
| Kidney | 20 | 7,464 |
| Gut | 14 | 6,933 |
| Thyroid | 20 | 5,756 |
| Testis | 20 | 8,673 |
| Muscle | 18 | 5,347 |
| Liver | 20 | 6,151 |
| Lung | 14 | 9,048 |

**Table S2.** Number of genes with allele-specific expression.

|  | | | **FDR** | |
| --- | --- | --- | --- | --- |
| Tissue | ***q*<0.1** | ***q*<0.05** | |  |
| Spleen | 1057 | 884 | |  |
| Brain | 840 | 659 | |  |
| Heart | 729 | 582 | |  |
| Kidney | 1000 | 784 | |  |
| Gut | 750 | 639 | |  |
| Thyroid | 403 | 354 | |  |
| Testis | 1255 | 971 | |  |
| Muscle | 563 | 457 | |  |
| Liver | 956 | 814 | |  |
| Lung | 892 | 730 | |  |

**Table S3.** Spearman’s rank correlation coefficient between gene expression-related measures and dN/dS between *M. m. domesticus* and *M. caroli.*

|  | ***rho*** | ***p*** |
| --- | --- | --- |
| Average expression level across tissues | -0.24 | < 2.2e-16 |
| Expression IQR^1^ across tissues | -0.22 | < 2.2e-16 |
| Average connectivity across tissues | -0.22 | < 2.2e-16 |
| Connectivity IQR^1^ across tissues | -0.13 | < 2.2e-16 |
| Number of consensus modules | -0.21 | < 2.2e-16 |

^1^ Interquantile range, where IQR = Quantile 3 - Quantile 1

**Table S4.** The relationship between gene expression and connectivity within tissues.

| Tissue | *rho^1^* | *P* |
| --- | --- | --- |
| Thyroid | 0.31 | < 2.2e-16 |
| Lung | 0.38 | < 2.2e-16 |
| Spleen | 0.49 | < 2.2e-16 |
| Muscle | 0.48 | < 2.2e-16 |
| Brain | 0.55 | < 2.2e-16 |
| Testis | 0.55 | < 2.2e-16 |
| Kidney | 0.62 | < 2.2e-16 |
| Gut | 0.63 | < 2.2e-16 |
| Liver | 0.55 | < 2.2e-16 |
| Heart | 0.44 | < 2.2e-16 |

**^1^**Spearman’s rank correlation *rho*

**Table S5.** Pairwise comparisons of gene connectivity between tissues (Spearman’s rank correlation, *rho*).

|  | **Lung** | **Kidney** | **Muscle** | **Liver** | **Thyroid** | **Testis** | **Brain** | **Gut** | **Heart** | **Spleen** |
| --- | --- | --- | --- | --- | --- | --- | --- | --- | --- | --- |
| **Lung** |  | 0.25 | 0.18 | 0.12 | 0.2 | 0.13 | 0.14 | 0.17 | 0.24 | 0.14 |
| **Kidney** |  |  | 0.27 | 0.29 | 0.35 | 0.23 | 0.23 | 0.33 | 0.29 | 0.21 |
| **Muscle** |  |  |  | 0.21 | 0.3 | 0.13 | 0.17 | 0.23 | 0.28 | 0.13 |
| **Liver** |  |  |  |  | 0.25 | 0.06 | 0.13 | 0.24 | 0.25 | 0.14 |
| **Thyroid** |  |  |  |  |  | 0.16 | 0.17 | 0.27 | 0.4 | 0.17 |
| **Testis** |  |  |  |  |  |  | 0.15 | 0.15 | 0.13 | 0.12 |
| **Brain** |  |  |  |  |  |  |  | 0.15 | 0.16 | 0.14 |
| **Gut** |  |  |  |  |  |  |  |  | 0.23 | 0.19 |
| **Heart** |  |  |  |  |  |  |  |  |  | 0.18 |

**Table S6.** Tissue-specific transcription factors are enriched for tissue-specific mutant phenotypes.

| Tissue | Tissue-specific mutant phenotypes | *q*-value |
| --- | --- | --- |
| Brain | Abnormal brain size | 1.725 x 10^-4^ |
|  | Abnormal brain weight | 0.003 |
|  | Abnormal cerebellar cortex morphology | 1 x 10^-3^ |
| Testis | Abnormal testis weight | 0.027 |
|  | Small testis | 0.01 |
|  | Abnormal seminiferous tubule size | 0.016 |
| Liver | Abnormal liver morphology | 0.01 |
|  | Abnormal liver size | 0.018 |
| Spleen | Abnormal spleen size | 0.002 |
|  | Small spleen | 0.002 |
|  | Enlarged Spleen | 0.017 |
|  | Abnormal splenocyte apoptosis | 0.004 |
|  | Abnormal spleen physiology | 0.006 |
|  | Abnormal splenocyte physiology | 0.002 |
|  | Abnormal splenocyte proliferation | 0.001 |

**Figure S1.** (**A**) The number of genes in each tissue that were classified as tissue-specific. In orange are genes that encode transcription factors. (**B**) The number of hub genes that are found across different numbers of tissues.

**Figure S2.** (**A**) Average expression across tissues and *S*_max_ are negatively correlated. (**B**) Connectivity across tissues is negatively correlated with *S*_max._

**Figure S3.** Genes for which we could detect allele-specific expression have higher expression on average (permutation test, *p*<0.0001).
